# Thomas Chevalier

**Published:** 1824-07

**Authors:** 


### OBITUARY.

On Wednesday, the 9th of June, at his house in South Audley-street, Thomas Chevalier, Esq. F.R.S. &c. &c. Surgeon Extraordinary to the King, and Professor of Anatomy and Surgery to the Royal College of Surgeons, London. Although Mr. Chevalier had for upwards of two years been suffering from severe and repeated attacks of indisposition, his death was unexpected and sudden; having been engaged in the active exercise of his profession the day previous to his dissolution. As a zealous and skilful practitioner, Mr. Chevalier was universally esteemed, and respected by his professional brethren. His general and professional reading was extensive; his ardour in the pursuit of knowledge, great; and the high character which he had attained was the deserved reward of a life devoted to the cultivation of science. Mr. C. was among the first, if not the very first, of those who obtained the Jacksonian Prize of the College of Surgeons. His Hunterian Oration may be mentioned as almost the only one of those compositions that has been read at all, or read with pleasure and advantage. He was likewise the author of a little treatise on Gun-shot Wounds, published at an early period of his professional career, and of many papers on various chirurgical subjects, interspersed in the periodical publications or in the Transactions of various learned Societies. If the Lectures delivered by Mr. C. at the College of Surgeons, and the volume lately published by him, fell somewhat short of the expectations that were formed by the profession, it must be recollected that those very expectations evinced the sense generally entertained of the merits of this gentleman, and that they were composed under the pressure of extreme ill health.

In private life, Mr. Chevalier's character stands in no need of any eulogium from us: he was generally respected, and will long live in the memory of his numerous friends. We understand that extravasation of blood in the brain was the sole and immediate cause of his death; though we have reason to believe that he had long entertained an opinion that there was disease, either of the great vessels or of the heart itself.

